# Integrating Cervical Cancer Screening with HIV Care in Cameroon: Comparative Risk Analysis of Cervical Disease in HIV-Infected Women Receiving Antiretroviral Therapy to Women in the General Population

**DOI:** 10.1371/journal.pone.0149152

**Published:** 2016-02-11

**Authors:** Cavin Epie Bekolo, Gillian O’Bryan, François Edmond Tchago, Charlette Nangue, Patrick Sylvestre Bekoule, Basile Kollo

**Affiliations:** 1 Centre Médical d’Arrondissement de Baré, Nkongsamba, Cameroon; 2 International Training & Education Center for Health (I-TECH), Seattle, United States of America; 3 Regional Hospital of Nkongsamba, Nkongsamba, Cameroon; 4 University Teaching Hospital, Yaounde, Cameroon; 5 Department of Public Health, University of Douala, Douala, Cameroon; Bharathidasan University, INDIA

## Abstract

**Background:**

While the effect of highly active antiretroviral therapy (HAART) on natural history of cervical lesions remains controversial, resource limited countries need to understand the relevance of their own data to their settings. We compared the risk of cervical disease in HAART-experienced women with that in women in the general population of Cameroon.

**Methods:**

A retrospective cross sectional survey of women aged 35 years and above, attending a voluntary screening campaign for cervical cancer at the Nkongsamba Regional Hospital in Cameroon between February and May 2014. Squamous intraepithelial lesions (SIL) were determined by Pap smear. Multiple logistic regression was used to compare the odds of SIL in women on HAART to women from the community with unknown HIV status.

**Results:**

Included were 302 women of whom 131(43.4%) were HIV-infected and receiving HAART on the site while 171 (56.6%) were women from the community. Cervical disease was observed in 51(16.9%) persons of whom 15 (11.5%) cases in the HAART group and 36 (21.1%) cases in the general group (p = 0.027). After controlling for age and other covariates, women in the HAART group had a 67% reduction in the odds of cervical lesions compared with the community group [adjusted odd ratio (aOR) = 0.33, 95%CI: 0.15–0.73, p = 0.006).

**Conclusion:**

HIV-infected women receiving HAART have a lower risk of cancer than women in the general population. This finding may not be attributed to HAART alone but to all the health benefits derived from receiving a comprehensive HIV care.

## Introduction

Cervical cancer is a preventable disease and is curable if diagnosed and treated early. Women living with human immunodeficiency virus (HIV) have a higher risk of human papilloma virus (HPV) infection, pre-cancer and invasive cervical cancer and should be screened and followed closely for evidence of pre-cancerous changes in the cervix, regardless of age, antiretroviral therapy (ART) status or CD4 count and viral load[1, 2]. Cervical cancer screening coupled with immediate management leads to early detection of precancerous and cancerous cervical lesions thus preventing serious morbidity and mortality. WHO guidance covers human papillomavirus vaccination and prevention, screening and treatment and palliative care of cervical cancer[3].

Though highly active antiretroviral therapy (HAART) has had an unequivocally positive impact on morbidity and mortality in HIV-infected individuals, the impact of HAART on cervical cancer, however, remains uncertain and conflicting[4, 5]. Published studies differ in their study designs, screening and diagnostic protocols, duration and type of HAART use, recruitment and referral strategies, and definitions of screening test and disease positivity. Innovative approach including quasi-experimental trials and operations research in sentinel populations to answer the critical research questions in this area have been suggested as possible solutions[5].

Access to HAART is expanding exponentially but while the debate on the effect of HAART on cervical diseases goes on, there is the necessity for each setting to consider the implications of their own data especially for resource limited settings in which cervical disease screening is rare in the general population and in women living with HIV (WLWHIV) in particular. In Cameroon, only 2.3% of all women aged 15–50 were ever screened for cervical cancer and there are no guidelines for cancer screening in WLWHIV. The crude incidence rate of cancer of the cervix uteri in Cameroon is estimated at 19.4 per 100000 with 1993 cases and 1120 deaths recorded annually [6]. In 2008, the prevalence of squamous intraepithelial lesions (SIL) in women on antiretroviral therapy in an urban Cameroon population was reported to be as high as 43.5% underscoring the need for screening and care in this population[7]. Access to HAART at this era was still very constraint. In this paper, by integrating cervical cancer screening into HIV care, were sought not only to reassess the condition in an era of increased access to HAART, but also to compare its prevalence and risk to that in the general female population as the control group. This approach would contribute not only to improving knowledge in the ongoing controversy concerning the impact of HAART on cervical disease, but would also help in redefining recommendations for the care of WLWHIV in the context of Cameroon.

## Methods

### Setting

The study was conducted at the Regional Hospital of Nkongsamba, Moungo Division of the Littoral Region of Cameroon. It is a second level reference public health facility with a catchment area of over 321,295 inhabitants [8]. The onsite HIV clinic was established in 2005 and offers HIV counselling and testing, ART and limited community outreach services to patients on ART. Screening for cervical disease in WLWHIV is not common practice.

### Ethical consideration

The study was approved by the medical council of the Nkongsamba Regional Hospital. Permission to use data was granted by the directorate of the Nkongsamba Regional Hospital, the custodian of the database. Individual participant consent could not be obtained because we were using patients’ records for this study. All patient information was anonymised and de-identified prior to analysis.

### Population and enrolment

Between February 26 and May 7, 2014 Nkongsamba Regional Hospital in Cameroon, held a voluntary cervical cancer screening campaign in honour of the International Women’s Day, an extension of its regular cervical cancer screening program. Free cervical disease screening was provided by a Peace Corps Cameroon Volunteer Activities Support and Training (VAST) grant. This voluntary screening was open to all women in the municipality. HIV positive women receiving treatment at the HIV clinic of Nkongsamba Regional Hospital as well as women from the community with unknown HIV status were eligible to participate in the screening if they were over the age of 35, not pregnant, and not severely ill. Women who were menstruating at enrolment were asked to return in one week to participate. Health workers screened potential eligible women and completed a brief intake questionnaire after verbally obtaining their consent. They collected information on socio-demographic characteristics, HIV and HAART statuses, lifestyle factors, reproductive characteristics, and family history of cancer (S1 File).

### Procedures

Each woman was screened using three different methods in series: visual inspection with acetic acid (VIA), visual inspection with Lugol’s iodine (VILI) and conventional Pap smear cytology. During a pelvic examination, visual appearance of the cervix was recorded before application of acetic acid or Lugol’s iodine at The Nkongsamba Regional Hospital. VIA was performed by applying 3–5% acetic acid solution to the cervix and waiting for three minutes. VIA was considered positive with the presence of well-defined acetowhite areas with sharp borders. After VIA screening VILI was performed by applying Lugol’s iodine solution to the cervix. Similarly, results were interpreted by a laboratory technician after waiting for another three minutes. VILI was considered positive with the presence of areas of non-uptake, which appear pale yellowish-white. VIA and VILI results were interpreted by the same laboratory technician. Following VIA and VILI screening a traditional Pap smear was performed with cervical cells collected and smeared onto slides, which were read and analysed by a pathologist at The University Teaching Hospital in Yaounde, Cameroon. The pathologist was blinded to results from the direct visual inspection (DVI). Women were referred to immediate biopsy if they had high-grade squamous intra-epithelial lesions (HSIL), atypical squamous cells cannot exclude high-grade lesion (ASC-H), or atypical glandular cells of undetermined significance (AGU-S). Women with low-grade squamous intra-epithelial lesions (LSIL) and atypical squamous cells of undetermined significance (ASCUS) were referred to repeat Pap smear in six months.

### Statistical analysis

Data analyses were performed using Stata^®^ 13.1 (StataCorp LP, Texas 77845, USA). A comparison of characteristics between WLWHIV on HAART and women from the general community was done. The main outcome of interest was cervical disease defined as the presence or absence of squamous intra-epithelial lesions (SIL) of any grade according to Pap smear cytology. The impact of HAART on human papilloma virus-related disease remains more clearly observable when evaluating SIL of any grade as the primary outcome[9]. Cervical cytology has been chosen by convention to define cervical lesion and was performed at a University Teaching Hospital, a first level of reference health facility. The characteristics compared were age, place of residence, occupation, religion, level of education, marital status, smoking habit, past pregnancy and childbearing history, and family history of cancer. Summary statistics were presented as proportions for categorical variables and as means (with standard deviations) for normal continuous variables or medians (with IQR—interquartile range) for skewed continuous variables. Pearson chi-squared test or Fisher exact tests for small samples were used where appropriate to assess for the association between categorical variables. The student t-test was used to test for the mean difference between two groups for continuous variables. A univariable logistic regression model was set up for the association between each exposure variable with cervical disease. Crude odd ratios, their 95% confidence intervals and p-values were reported. Factors associated with cervical disease at 5% significance level, were included in a multiple logistic regression model. Backwards elimination based on p-value was used to pick up factors independently associated with cervical lesion. The corresponding adjusted odds ratios, their 95% confidence intervals and p-values in the final model were reported.

## Results

Included were 302 women of whom 131 (43.4%) were receiving HAART and 171 (56.6%) were women from the community with unknown HIV status. Their median age was 47 (IQR: 42–55) years but women from the community were on average two years older than women on HAART (49.7 ± 8.6 versus 47.7 ± 8.3 years, p = 0.01). Women from the general community had a better experience of cervical cancer screening than women on HAART (16.1% vs 3.8%, p = 0.001) and were more likely to report a family history of cancer (24.4% vs 11.5%, p = 0.005). Women from the community also showed significant differences from women on HAART in terms of education, occupation, place of origin and marital status (Table 1). The two groups were similar in their religious beliefs, smoking habits as well as in their previous history of pregnancies and childbearing potentials.

**Table 1:** Comparison of baseline characteristics of the study groups.

Features	Community group (n = 171)	HAART group (n = 131)	p-value for the difference
**Age in years mean(SD)**	**49.7(8.6)**	**47.1(8.3)**	**0.01**
**Occupation, n (%)**			
Housewife	127 (74.3)	104 (79.4)	
Teacher	29 (17.0)	1 (0.8)	
Other	15 (8.7)	26 (19.8)	
**Total**	**171 (100)**	**131 (100)**	**<0.001**
**Region of origin, n (%)**			
West	115 (67.3)	65 (50.0)	
Littoral	44 (25.7)	45 (34.6)	
Other	12 (7.0)	20 (15.4)	
**Total**	**171 (100)**	**130 (100)**	**0.005**
**Religion, n (%)**			
Catholic	79 (46.2)	57 (43.5)	
Protestant	71 (41.5)	50 (38.2)	
Muslim	4 (2.3)	3 (2.3)	
Other	17 (9.9)	21 (16.0)	
**Total**	**171 (100)**	**131 (100)**	**0.473**
**Marital status, n (%)**			
Married	125 (73.1)	50 (38.1)	
Single	22 (12.9)	36 (27.5)	
Widowed	24 (14.0)	44 (33.6)	
Divorced	0 (0.0)	1 (0.8)	
**Total**	**171 (100)**	**131 (100)**	**<0.001**
**Education, n (%)**			
None	9 (5.3)	13 (9.9)	
Primary	51 (29.9)	67 (51.2)	
Secondary	107 (62.6)	49 (37.4)	
Higher	4 (2.3)	2 (1.5)	
**Total**	**171 (100)**	**131 (100)**	**<0.001**
Gravidity*			
> 5	77 (45.3)	61 (46.9)	
≤ 5	93 (54.7)	69 (53.1)	
**Total**	**170 (100)**	**130 (100)**	**0.779**
Parity*			
> 5	58 (34.1)	36 (27.7)	
≤ 5	112 (65.9)	94 (72.3)	
**Total**	**170 (100)**	**130 (100)**	**0.234**
Currently smoking*			
No	167 (98.8)	126 (96.9)	
Yes	2 (1.2)	4 (3.1)	
**Total**	**169 (100)**	**130 (100)**	**0.247**
Family history of cancer*			
No	127 (75.6)	115 (88.5)	
Yes	41 (24.4)	15 (11.5)	
**Total**	**168 (100)**	**130 (100)**	**0.005**
Previous screening*			
Never	141 (83.9)	125 (96.2)	
Ever	27 (16.1)	5 (3.8)	
**Total**	**168 (100)**	**130 (100)**	**0.001**

*Missing data in these variables

**Fig 1:**
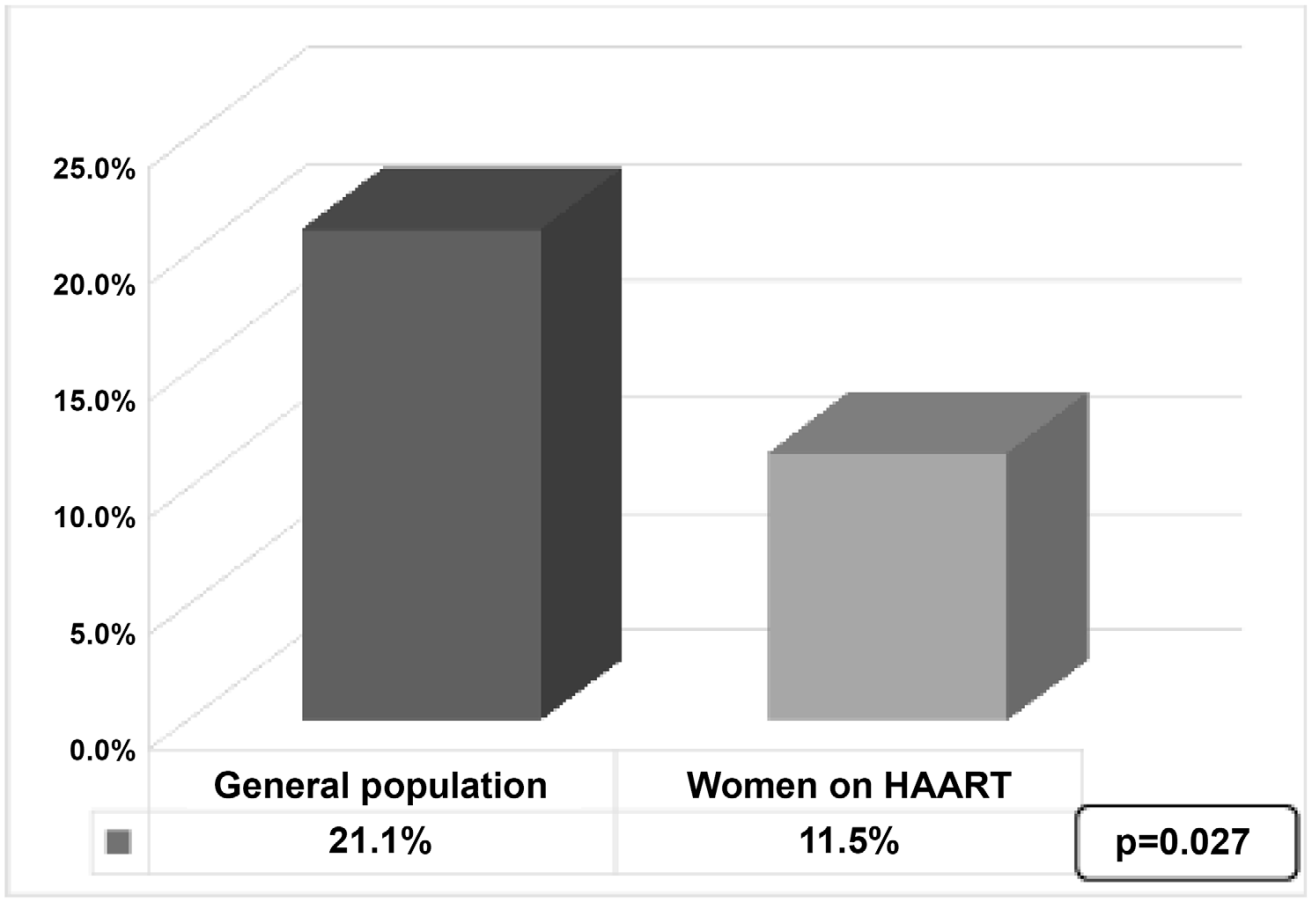
Prevalence of abnormal Pap smear cytology in the study groups.

Direct visual inspection (DVI) with VIA showed abnormal results in 40 (13.4%) women of whom 24 were in the community group and 16 (12.3%) in the HAART group (p = 0.634). The sensitivity and specificity of VIA was 6% and 85% respectively using Pap smear as gold standard (kappa = -0.05). DVI with VILI revealed abnormal lesions in 60 (20.2%) women of whom 33 (19.8%) from the community group and 27 (20.8) from the HAART group (p = 0.83). The sensitivity and specificity of VILI was 10% and 78% respectively using Pap smear as gold standard (kappa = -0.06). There was good agreement between results reported for VIA and VILI (kappa = 0.68). Abnormal Pap smear (SIL) results were reported in 51 women corresponding to an overall prevalence of cervical disease of 16.9%.Of these women with abnormal cytology, LSIL was observed in 19 (6.3%), ASCUS in 7 (2.3%), ASC-H and AGU-S in 4 (1.3%) and HSIL in 21 (7.0%) of them. Fig 1 shows that there was a significantly higher proportion of women from the general community 36 (21.1%) than women receiving HAART women 15 (11.5%) who presented with cervical lesion (p = 0.027).

After controlling for the differences in characteristics between the two groups, we observed a two-third reduction in the odds of cervical disease in the HAART group (aOR = 0.33, 95%CI: 0.15–0.72, p = 0.006) compared with the community group. Age was also an independent risk factor for cervical disease as women aged 55 years old and above were associated with a three-fold increase in the risk of cervical disease (Table 2).

**Table 2:** Logistic regression model of factors associated with cervical disease.

Factor	Cervical lesion n (%)	OR (95%CI)	p-value	aOR (95%CI)	p-value
**Group**					
Community	36 (21.1)	1		1	
HAART	15 (11.5)	0.48 (0.25–0.93)	0.029	0.33 (0.15–0.72)	0.006
**Age group in years**					
< 55	22 (12.2)	1		1	
≥ 55	29 (24.0)	2.28 (1.24–4.20)	0.008	3.01 (1.36–6.69)	0.007

OR: crude odd ratio, aOR: adjusted odd ratio, CI: confidence interval

## Discussion

This study has indicated that it may be possible to link up HIV care to cervical screening in a resource limited setting and that women in HIV care and receiving HAART had a risk of cervical lesions that was two-thirds lower than their counterparts in the general population in Cameroon.

### Integrating cancer screening program with HIV care

Although access to HAART has been rolled out in the recent decade, the integration of cervical cancer screening with HIV care is rare or inexistent in most low and middle income countries as a result of persistent lack of resources and competing priorities in ensuring a sustainable and comprehensive HIV care. By adopting and successfully implementing for three successive years, the “see-and-treat-or-refer” approach for cervical cancer control, a level two referral health facility in a predominantly rural Cameroonian setting, has demonstrated that, it is feasible to nest a cancer control program into healthcare in general and HIV care in particular. The health facility provided the resources needed to implement the program with the low cost VIA/VILI technology but its extension to the more expensive cervical cytology was made possible by a Peace Corps Cameroon Volunteer Activities Support and Training (VAST) grant. This is an illustration of a pragmatic public-private partnership initiated at the operational level of a health system in which health programs are more vertical than horizontal in practice. Health facilities and HIV programs could be encouraged to take similar initiatives by making use of their own resources but their efforts need to be supplemented by support from other sources. HIV programs in LMICs are currently gaining substantial support from partners like the Global Fund and PEPFAR (President’s Emergency Plan for AIDS Relief) to help scale up the screening program [10].

While it might be feasible to broaden an HIV program to include cancer screening, such a screening would be of less relevance and unsuccessful according to the principles of Wilson & Jungner [11], if post-screening follow-up, treatment or referral systems are not effective. Women in HIV care can be traced and followed up while receiving free HAART but they cannot be provided treatment for cervical lesions in HIV clinics. They need to seek care in tertiary health facilities by paying out of their pockets. To date, we have little or no idea about the outcome of women who were referred for confirmatory diagnostic investigations and subsequent treatment. Women with a ‘false positive’ results after screening may suffer from psychological disturbances if they cannot have a confirmatory test done. Similarly, those who require treatment but cannot afford would consider screening as a ‘necessary evil’. These barriers to a successful screening program and thus its linkage to HIV care are likely to annihilate achievements made so far and thus discourage further efforts.

The screening program employed three different methods including VIA, VILI and the conventional Pap smear. While VIA and VILI techniques showed good agreement in detecting cervical abnormalities, there was considerable discrepancy between the DVI techniques and Pap smear. The DVI procedures were performed in the same centre and by the team of nurses who also collected, smeared, stored and transported cell samples for cytology. The cytology results were read and interpreted by a trained pathologist at a tertiary hospital. The pathologist was blinded to DVI results. None of these procedures or interpretation of results were double-checked to ensure quality assurance (QA). This practice is a reflection of the routine clinical procedure in the health facility. Lack of QA may be responsible for the observed disagreement between DVI techniques and Pap smear. The impact of the discrepancy on individual patients and the screening program is negligible because follow up visits and confirmatory diagnostic tests can correct for false results. However, for the purpose of research, inadequate QA may introduce systematic errors in our findings. To reduce this bias, we used SIL of any grade as our primary outcome[9, 12] and because the cervical cytology was performed in a first level of referral hospital in which we have a higher degree of trust.

### HAART and evolution of cervical disease prevalence in Cameroon

The 11.5% prevalence of SIL in women receiving HAART in this study was much lower than the 48.6% and 43.5% prevalence rates observed in 2009 and 2008 respectively in women initiating HAART in similar settings of urban Cameroon [7, 13]. This is an indication that cervical disease prevalence has decreased over time. Women in the earlier cohorts had low median CD4 counts of 253.7 cells/μl and 197cells/μl respectively while women in our cohort were HAART-experienced and supposedly had a median CD4 of about 436 cells/μl [14].This relatively low prevalence can thus be associated with an increased regression and decreased incidence of human papillomavirus (HPV)-related cervical lesions among HIV-infected women on HAART [12, 15] owing to improved levels of immune status[16]; and to the improved access to HAART and retention in HIV care in Cameroon during this period of time [17, 18]. Our findings may thus be tilting in favour of reduced prevalence of cervical disease in the era of HAART scale up despite increased survival. We could not however, account for differences in methods and quality assurance in the ascertainment of cervical lesions as well as other risk factors for cervical cancer that must have changed over time. As a result, it is difficult to attribute the observed decrease in prevalence of SIL to HAART alone.

### HAART and risk of cervical disease

It is known that women infected with HIV have a significantly elevated risk of cervical cancer and its precursors when compared with the general population[19, 20]. We speculated that HAART by partially restoring the immune status will reduce the risk of cervical disease relative to HAART-naïve HIV-infected women, but by prolonging life HAART will also increase the risk as a result of ageing and prolonged exposure to oncogenic factors. The overall risk was expected to remain higher or at most similar to women in the general population. Current evidence about the effect of HAART on cervical cancer risk is mixed and had compared HAART and non-HAART groups[5]. Our findings suggest that the risk of cervical disease was much lower in women receiving HAART compared with women in the general population even after controlling for known differences between the two groups. Though the findings may be internally valid for HIV-infected women, there is no reason at this moment why we should think that HAART per se is protective against cervical disease in the general population even though HAART may provide some protection against HPV [21–23], the main risk factor for cervical cancer, it has never been tested for this purpose. Yet we want to imagine that by assuming that both groups had similar risk of cervical disease as “healthy volunteers” some unmeasured but predictable differences between the groups may have also been responsible for the low risk of SIL in the HAART group. A supposedly change in risky lifestyles such as cessation of smoking, moderation of alcohol intake, restriction of sexual partners and safer sex practices adopted more by women in HAART[24, 25] as they are taught during their regular visits to the HIV clinic for continuous care, may have reduced their risk of developing cervical disease. This study therefore indicates that HAART is directly or indirectly via comprehensive HIV care associated with a reduction in the odds of cervical cancer. However, we want to warn against the threat of relaxing its cancer screening in women on HAART because as they live longer and especially after the age of 55 years, the risk triples.

Our study had some limitations. The Pap smears lacked quality assurance but there is no indication that there was a differential interpretation of results between the HAART group and the community group because the pathologist was blinded to the participants’ information. The observational design cannot provide a causal relationship between HAART and cervical disease but has provided an additional argument in favour of a protective effect of HAART in HIV-infected women. The use of hormonal contraception, the number of sexual partners and HPV vaccination has not been accounted for.

## Conclusions

In conclusion, this study suggested that it is feasible to integrate cervical cancer screening with HIV care and that HAART has been associated with a reduction in the risk and burden of cervical disease in HIV-infected women in Cameroon. The controversy on effect of HAART on cervical lesions has not changed significantly at the global stage as a result of these findings but in a setting with a high burden of HIV and cervical cancer, HAART is changing the status quo.

## Supporting Information

S1 File The coded dataset. (XLSX)
